# ESBL-plasmid carriage in *E. coli* enhances in vitro bacterial competition fitness and serum resistance in some strains of pandemic sequence types without overall fitness cost

**DOI:** 10.1186/s13099-018-0243-z

**Published:** 2018-06-15

**Authors:** Amit Ranjan, Julia Scholz, Torsten Semmler, Lothar H. Wieler, Christa Ewers, Stefanie Müller, Derek J. Pickard, Peter Schierack, Karsten Tedin, Niyaz Ahmed, Katharina Schaufler, Sebastian Guenther

**Affiliations:** 1 Institute of Microbiology and Epizootics, Veterinary Faculty, Freie Universität Berlin, Robert-von-Ostertag Str. 7-13, 14163 Berlin, Germany; 2 Pathogen Biology Laboratory, Department of Biotechnology and Bioinformatics, School of Life Sciences, University of Hyderabad, Hyderabad, India; 3 Robert Koch Institute, Berlin, Germany; 4 Institute of Hygiene and Infectious Diseases of Animals, Veterinary Faculty, Justus-Liebig-Universität Giessen, Giessen, Germany; 5 Welcome Trust Sanger Institute, Cambridge, UK; 6 Brandenburgische Technische Universität Cottbus-Senftenberg, Cottbus, Germany; 7 International Centre for Diarrheal Disease Research Bangladesh (icddr,b), Mohakhali, Dhaka, Bangladesh; 8 Institute of Pharmacy, Pharmaceutical Biology, University of Greifswald, Greifswald, Germany

## Abstract

**Background:**

Extended spectrum beta lactamase (ESBL)-producing extraintestinal pathogenic *Escherichia coli* infections are of global interest because of their clinical and economic impact. The ESBL resistance genes disseminate through plasmids, and are found in successful global lineages such as ST131 and ST648. The carriage of plasmids has been suggested to result in a fitness burden, but recently it was shown that ESBL-plasmids enhanced virulence in pandemic ST131 and ST648 lineages without affecting their fitness. Herein, we investigated the influence of ESBL-plasmids on bacterial competition and serum resistance, both of which are essential characteristics of ExPEC during infections.

**Methods:**

Triplets of ESBL-plasmid-carrying wildtype (WT), plasmid-cured variant (PCV) and transformant (T) of five ExPEC strains of ST131 and ST648 were used for bacterial competition experiments with colicin-producing commensal *E. coli*, competitive adhesion experiments and serum survival. In addition, resilience after SDS, acid, osmotic challenges and RNA sequence data were analyzed.

**Results:**

In all five strains tested, ESBL-plasmid carriage did not negatively influence *E. coli* fitness in direct bacterial competition with commensal *E. coli* in vitro. That is, WTs did not show any disadvantages when compared to their isogenic plasmid-free PCV. For one strain we even found the opposite as PCV17433 was out-competed by a commensal strain, which suggests an even protective role of the ESBL-plasmid carried by the WT17433. Similarly, in the serum-resistance experiments, the PCVs of two strains (PCV17433 and PCV17887) were more sensitive to serum, unlike WTs and Ts. The observed inter-strain differences could be explained by the different genetic content of plasmids carried in those strains.

**Conclusions:**

Overall, we found no compelling evidence for an increased burden resulting from the carriage of ESBL-plasmids in the absence of antimicrobial selection pressure in the strains of pandemic ST131 and ST648; rather, the possession of certain ESBL-plasmids was beneficial for some strains in regarding competition fitness and serum survival.

**Electronic supplementary material:**

The online version of this article (10.1186/s13099-018-0243-z) contains supplementary material, which is available to authorized users.

## Background

*Escherichia coli* (*E. coli*) are among the most common pathogens of extra-intestinal (ExPEC) and intestinal infections (InPEC) in humans and animals [1, 2]. ExPEC cause urinary tract, bloodstream, skin, and soft tissue infections as well as meningitis in humans and are causative agents for colisepticemia and other infections in poultry [1, 3, 4]. Treatment of ExPEC with antimicrobial agents results in an significant economic burden to healthcare facilities and in the poultry industry [5]. In recent years, an increase in resistance towards antimicrobials, including third generation cephalosporins, due to the production of extended-spectrum beta-lactamases (ESBL), has reduced treatment possibilities [6]. Dissemination of ESBL occurs not only through resistance plasmids, in addition certain clones referred to as “high risk clones” (HRC) such as ST131 and ST648 are also responsible for the spread across different niches [7, 8]. ST131 is the predominant clone isolated from *E. coli* infections reported worldwide, whereas ST648 is an emerging high risk clone reported in recent years from various habitats [3, 9–12]. For both clones, recent advances in their detection will likely further increase their identification as contributors to *E. coli* infections [13, 14].

Although ST131 (phylogroup B2) and ST648 (phylogroup D) differ in their phylogenetic background, both lineages show similar epidemiological success, and disseminate resistance traits equally in different populations [11, 15]. Both lineages also often host large ESBL-plasmids carrying CTX-M enzymes, which have also been found integrated into the chromosome [16, 17]. ESBL-plasmids have been reported to confer fitness costs for the host strain [18], but in addition to resistance factors, they also carry a large number of non-resistance genes. These “cargo” genes encode for variety of uncharacterized, hypothetical proteins, and their possible impact on fitness are yet to be explored [19]. Recently, we demonstrated that ESBL-plasmids do not reduce fitness in strains of these pandemic sequence types ST131 and ST648, but rather contributed to virulence in terms of biofilm formation in some strains through interactions between plasmid and host chromosomally-encoded factors [20]. This observation likely contributes to the success of certain HRC worldwide, including environments with low antimicrobial selection pressures.

The majority of ESBL-producing *E. coli* are ExPEC, capable of causing severe bloodstream infections. Thus, serum survival is an important virulence trait in those pathogens. In this study, we investigated whether *E. coli* wildtype isolates of two HRC clones, ST131 and ST648, which harbor ESBL-plasmids, demonstrate better serum survival when compared to their isogenic, ESBL-plasmid-free variant (PCV). As the reservoir of ExPEC is mainly the gut of humans and animals, intestinal epithelial adhesion is a preliminary step to colonization and systemic infection in competition with other bacteria. We performed competition assays with commensal, colicin-producing ST10 *E. coli* strains, alone and in combination with cell culture adhesion assays, to determine the overall competition fitness and adhesion with regard to the presence of the ESBL-plasmids.

## Methods

### Bacterial strains

Five wildtype ESBL-producing *E. coli* strains of two STs (ST131: IMT17433, IMT27685, IMT19205, ST648: IMT17887, IMT16316), their ESBL-plasmid-cured variants (PCV) and transformants (T) with a reintroduced ESBL-plasmid were analyzed in this study [21]. The isolates have been characterized in a previous publication [20, 21]. The details of plasmids and their properties are summarized in Additional file 1: Table S1. Strains and plasmids were chosen based on their original host, type of disease, size of the plasmid, CTX-M variant and inc/rep types, resulting in a broad spectrum of different combinations of plasmids with the bacterial chromosome. Two commensal strains of ST10 originally isolated from human gut [IMT13353 (colicin V, colicin M) and IMT13858 (colicin V, colicin E1), both resistant to chromosomally-encoded chloramphenicol] were used as colicin-producing commensal competitor strains. PCV19205 was not used for competition and competitive adhesion experiments as it was sensitive to the antibiotic enrofloxacin.

### Colicin extraction and activity assay

Colicin extraction was prepared as described earlier with modifications [22]. To test colicin activity, 5 ml of autoclaved 0.7% agar (close to solidifying temperature) was mixed with 60 μl of overnight-grown colicin-sensitive strains (*E. coli* BL-21-DE3 and MG1655) and poured onto LB agar plates. 5 μl of the extracted sample were added on top and incubated overnight at 37 °C. Zones of inhibited growth of the colicin-sensitive strains indicated colicin activity.

### Phenotypic microarray (PM)

The metabolic capability of the two colicin-producing ST10 competitor strains was determined using the BioLog Omnilog system and growth curves as described previously [20]. Six plates, PM1 to PM4, PM13 and PM14 (http://www.biolog.com/products-static/phenotype_microbial_cells_microPlate_panels_and_media.php) were used. PM1 to PM4 contain carbon, nitrogen, phosphorus and sulphur sources, PM13 and 14 contain antimicrobials and other chemical compounds. The plates were inoculated following the manufacturer’s instructions and grown for 48 h at 37 °C. The experiment was performed using three biological replicates. Data were analyzed using the statistics program R and heat maps were generated taking the area under curve (AUC) value for each well, normalized by the negative control wells [20, 23].

### Competition assay

Single competition assays between colicin-producing *E. coli* ST10 and the different variants of the ESBL-strains (WT, PCV or T) were performed. Experiments were repeated three times. Briefly, overnight cultures were grown to an OD_600_ = 1. One millilitre of each WT, PCV or T and the colicin-producing strain were added to 98 ml of LB medium and grown at 37 °C. At 60 min. intervals, 1 ml of culture was collected in duplicate. Following serial dilution, 100 μl of three different dilutions were used for differential plating. We used LB plates containing 4 µg/ml enrofloxacin (WT, PCV and T: enrofloxacin resistant and chloramphenicol sensitive), 32 μg/ml chloramphenicol (colicin-producing *E. coli* ST10: chloramphenicol resistant and enrofloxacin sensitive) and both (any possible transformation). Growth was determined by colony forming units (CFU) and graphs were plotted using GraphPad prism 5.01.

### Competitive adhesion experiments

Competitive adhesion experiments between colicin-producing *E. coli* ST10 and ESBL-triplets (WT, PCV and T) on porcine small intestinal (IPEC-J2) cell lines were performed as described elsewhere with modifications for differential plating [24]. The experiments were performed for each combination using three biological replicates and three technical replicates.

### Serum resistance experiments

Serum experiments were performed in human and chicken serum (Pan biotech, Germany) as described previously [24]. 5 μl of overnight grown culture were inoculated in 495 μl fresh LB medium and grown for 1.5 h. Bacteria were centrifuged at 7500×*g* for 3 min and resuspended in 1 ml of sterile 1X PBS. Thirty microliters were added in triplicates to 96-well microtiter plates containing 270 μl of 50% serum. Thirty microliters of sample were collected from each well, serially diluted and plated on LB plates (0 h count). The microtiter plates were incubated for 4 h at 37 °C. Following incubation, 30 μl of culture was plated as previously described (4 h count). Growth in serum was obtained by determining differences in the CFU after 4 h of incubation to that of 0 h. This experiment was performed with three technical and three biological replicates.

### RNA sequencing

Comparative RNA sequencing data of the WT, PCV and T of IMT17433 from our previous study was used to detect up- or down-regulated genes, which had been described to be involved in bacterial competition and serum resistance. Details on the RNA isolation procedure and gene regulation data analysis can be found in the original publication [20].

### Survival in SDS and under acidic and osmotic stress

Two strains triplets showing differences in bacterial competition and serum survival (WT/PCV/T17433, WT/PCV/T17887) and one control (WT/PCV/T16316) were used to analyze survival in 2% SDS, pH 3.8 and 5 M NaCl. The experiments were performed according to Wang [25].

### Statistical analysis

All statistical tests were performed using GraphPad Prism 5.01. Mann–Whitney U test was performed to calculate the p values for serum resistance and competitive adhesion experiments and values < 0.05 was considered as significant.

## Results

### Commensal strains produce colicin

Colicin production was detected as clear zones around the spots plated with colicin extract onto colicin-sensitive strains. Colicin extracts of both the competitor strains IMT13353 and IMT13858 showed clear zones on colicin-sensitive strains *E. coli* BL21 (λDE3) and MG1655, indicating the colicin production by these strains. Both the competitor strains IMT13353 and IMT13858 exhibited resistance to chloramphenicol which was chromosomally-encoded and used as selection marker for further experiments.

### Metabolic profiles among the competitor and wild type strains are comparable

To determine whether differences in metabolic activities might confer competitive disadvantages among the strains, we performed phenotypic microarray profiling using Omnilog plates containing different metabolites. We observed no significant differences in the metabolic profiles between the competitor strains IMT13353 and IMT13858, or any of the five wildtype ESBL strains for all four assays for carbon, nitrogen, phosphorous and sulphur sources (Additional file 1: Figure S1A–D), consistent with our previous study [20]. Thus, metabolic differences would not appear to contribute a significant role in possible phenotypes among competitor and wildtype strains.

### Competition reveals PCV17433 exceptional behavior against one ST10 strain

**Fig. 1:**
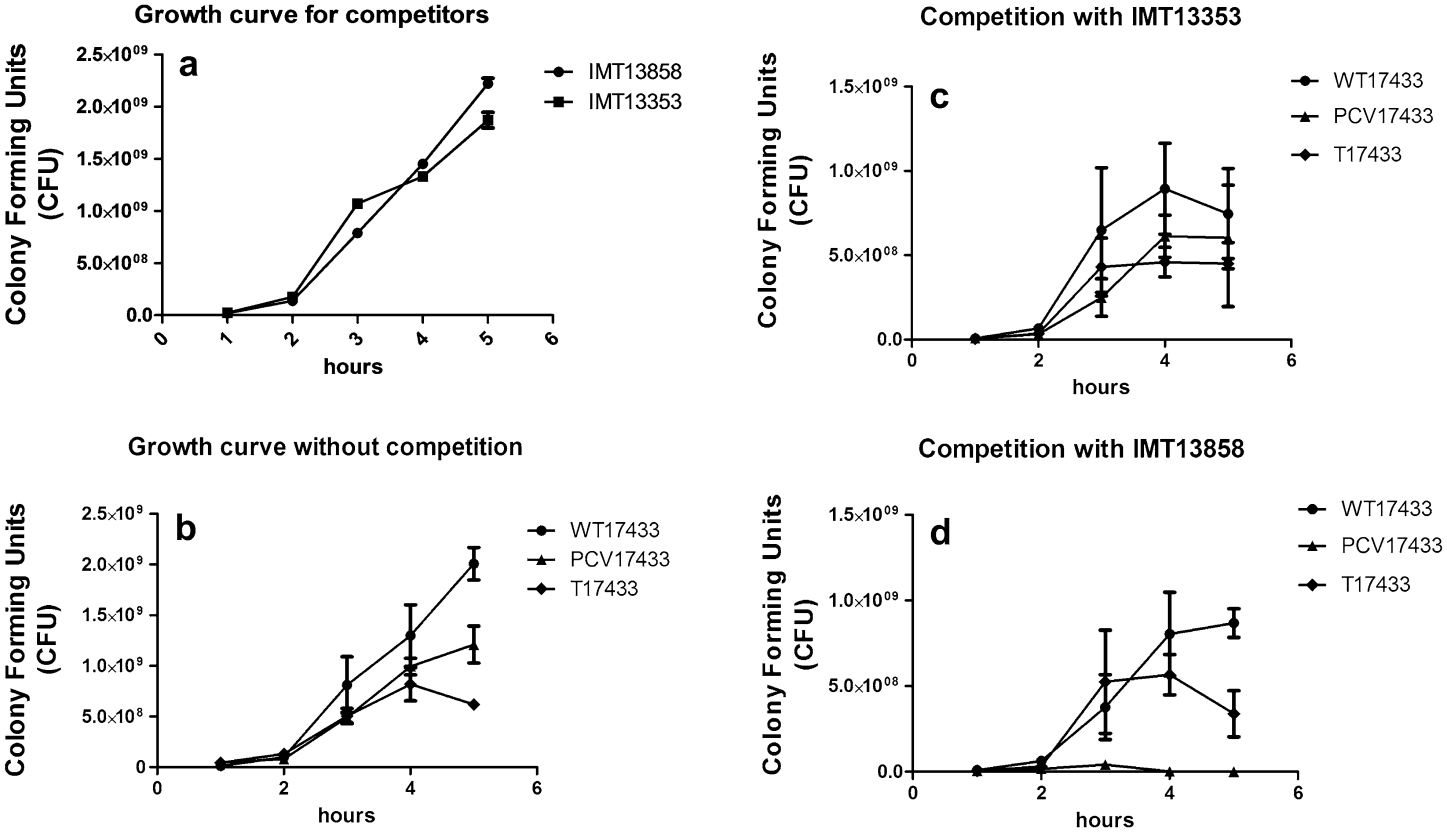
**a** Growth curve of both competitors, **b** growth curve of triplet of IMT17433 without competition and competition assay of the triplet of IMT17433 with competitor strain **c** IMT13353 and **d** IMT13858. Growth is measured in terms of colony forming units for a period of five hours

Both the ST10 colicin-producing commensal strains showed similar growth curves. In competitive growth assays, all three variants of the four strains IMT16316, IMT17433, IMT17887 and IMT27685 showed no differences in growth in the presence of the competitor strain IMT13353, and showed similar CFU counts for five hours of competition (Additional file 1: Figure S2A–F). However, in competitive growth assays with strain IMT13858, strain PCV17433 showed reduced growth, whereas for both the wildtype IMT17433 and plasmid-complemented strain, no differences were observed (Fig. 1). This exceptional behavior of PCV17433 suggested a possible role for the ESBL-plasmid in IMT17433 in protection against the colicin produced by IMT13858.

### PCV17433 (ST131) and PCV17887 (ST648) adhere significantly less in competitive adhesion with commensal strains

**Fig. 2:**
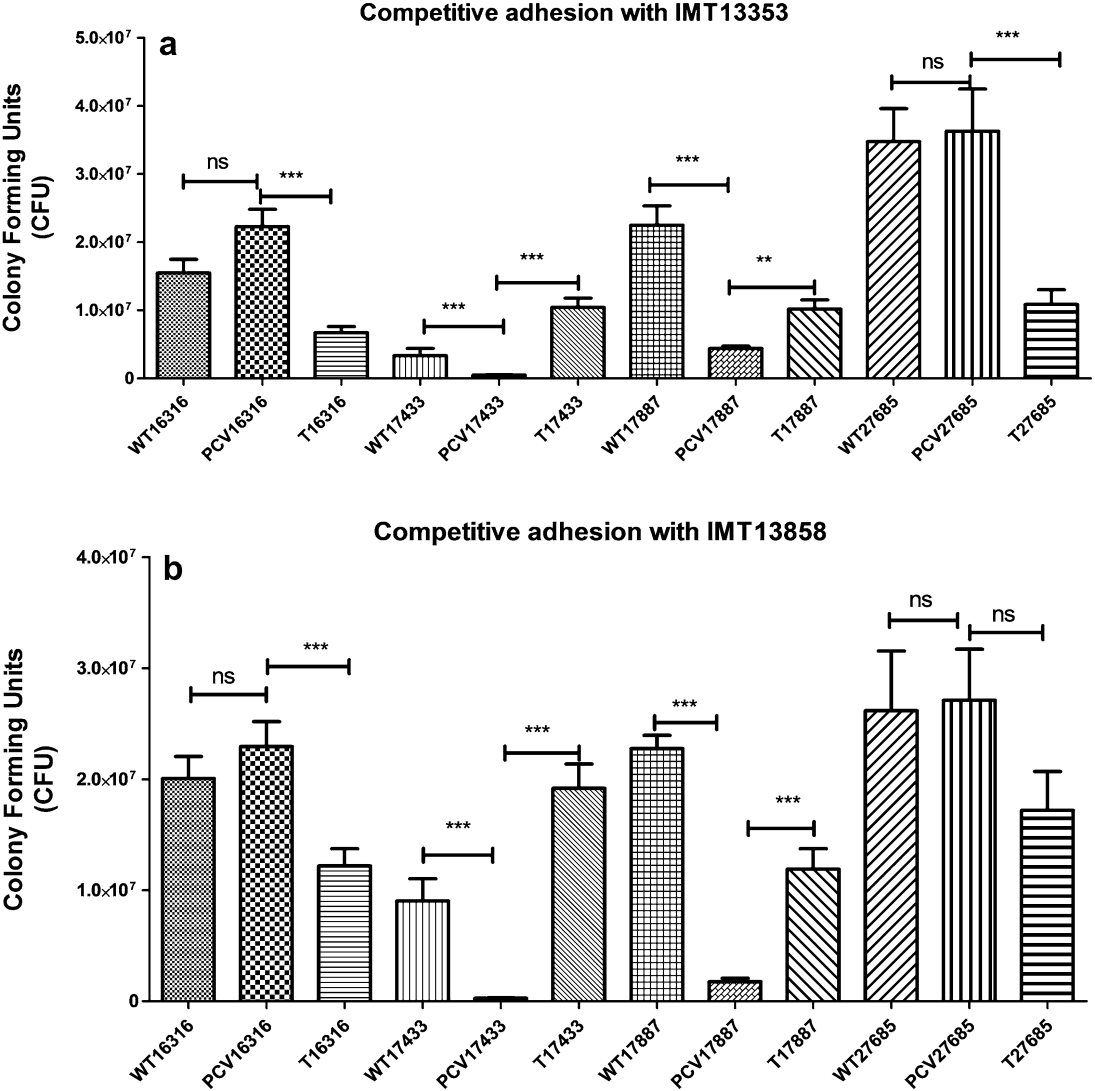
Competitive adhesion assay in IPEC-J2 cells with competitor strain **a** IMT13353 and **b** IMT13858. Growth is measured in terms of colony forming units. ns represents non significant *p* value while **p value ≤ 0.01 and ***p value ≤ 0.001

ExPEC strains are known to adhere to variety of epithelial cells. We therefore also performed competitive adhesion assays with both commensal ST10 strains against the four strains of HRC (ST131 and ST648) in the porcine, intestinal epithelial IPEC-J2 cell line. Strain PCV17433 was found to adhere significantly less than its variants in the adhesion competition assays. Likewise, strain PCV17887 showed a similar behavior against both the competitor strains IMT13353 and IMT13858 (Fig. 2a, b). These results suggested a role for the respective ESBL-plasmids in both adhesion in a competitive environment and protective effects against colicin-producing commensal strains.

### PCV17433 and PCV17887 are sensitive to different serum

**Fig. 3:**
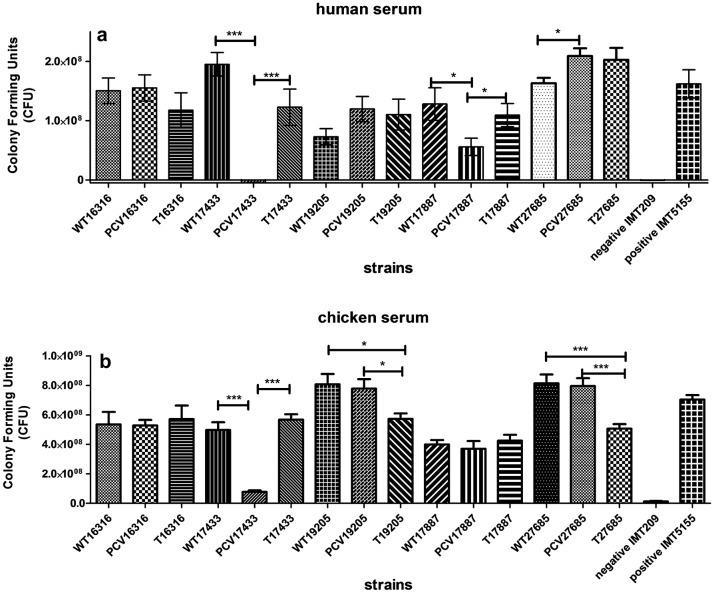
Serum bactericidal activity of triplets of all five strains in 50% **a** human serum, **b** chicken serum. IMT209 is used as negative control while IMT5155 as positive control for the assay. p values are represented by asterisk wherever significant as; *p value ≤ 0.05, **p value ≤ 0.01 and ***p value ≤ 0.001

Resistance to serum during infection is an important virulence property of ExPEC strains [26]. We therefore determined the resistance against both human and chicken serum. Consistent with prior observations, we found the plasmid-cured strain PCV17433 to be significantly more sensitive to both human as well as chicken serum (Fig. 3a, b). Interestingly, strain PCV17887 was sensitive to human serum but not to chicken serum. All five wildtype strains showed resistance to sera, and the plasmid-complemented (T) variants regained wildtype resistance, with few exceptions.

### Genes belonging to serum resistome are upregulated in WT strains

Resistance towards serum is complex and involves many different processes. The serum resistome has been described to comprise 56 essential genes or factors in a ST131 strain (EC958) [26]. Several of these factors, *nha, dnaJ, acrA* and *pgm*, were found to be upregulated by more than twofold in the wildtype strains of these same isolates in RNA seq data in our earlier study [20]. Furthermore, *ompA* was upregulated more than 2.4-fold compared to the PCV variant of the wildtype strain. OmpA is involved in serum resistance and has been described previously in *E. coli* K-12 [27]. These results suggest the ESBL-plasmids influence serum resistance in some strains of pandemic ST131 and ST648 isolates.

### PCV17433 and PCV17887 shows decreased survival in osmotic, salt and acid stress

**Fig. 4:**
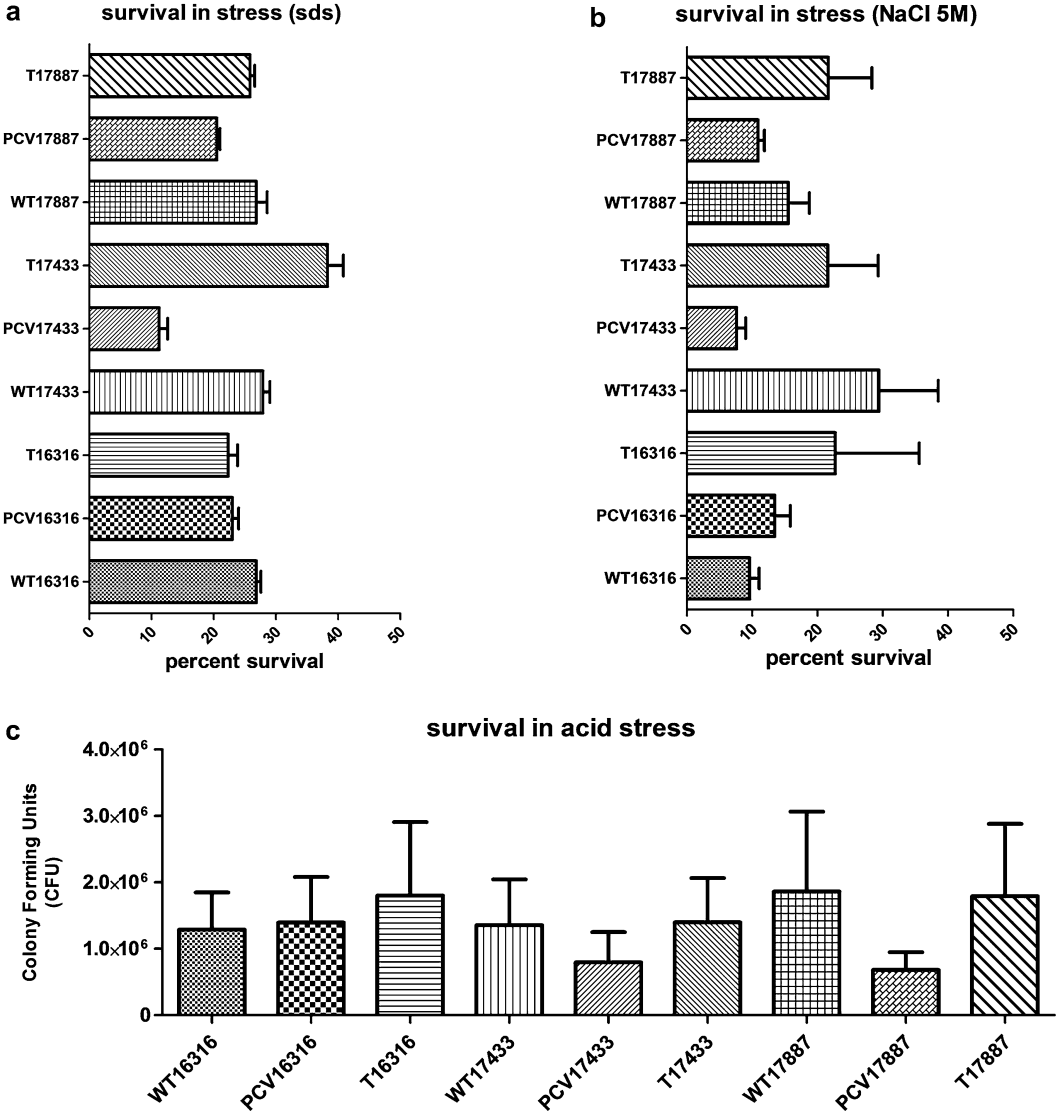
Growth of triplets in stress conditions **a** 2 M SDS, **b** 5 M NaCl and **c** Acetic acid. Survival in SDS and NaCl is represented in terms of percent survival while in acetic acid it is represented in terms of colony forming units (CFU)

Outer membrane proteins are additionally involved in a variety of functions related to stress in pathogenic organisms. We therefore determined whether the PCV variants also demonstrated differences in growth during stress conditions such as high salt (NaCl), SDS, and acid stress. As shown in (Fig. 4a–c), PCV17433 and PCV17887 both showed reduced survival compared to their WT and complemented (T) variant in all three stress conditions. No such changes were observed in control strain IMT16316 and its variants.

## Discussion

The emergence and dissemination of ESBL-producing *E. coli* are of global concern, as treatment opportunities are now restricted to the use of last resort antibiotics such as carbapenems [28]. This situation has become more precarious, as an increase in carbapenem-resistant strains has been reported worldwide [28, 29]. Although the dissemination is largely driven by resistance plasmids, high risk clones such as ST131 or ST648 also play an important role, as they represent successful lineages which dominate clinical cases across different host and sites [11, 12].

A large number of hypothetical genes from these large plasmids remain unexplored, and their implications with regard to clinical or virulence attributes are still unknown. In a previous study we found that ESBL-plasmids enhance biofilm formation in ST131 and ST648 *E. coli* [20]. Here, we explored possible impact of different ESBL-plasmids on important virulence characteristics such as competition with commensal microorganisms, and serum-resistance. Five different strains harboring different plasmids (Additional file 1) were chosen based on their original host type, disease association, size of the plasmid, CTX-M variant and inc/rep types. We examined different combinations of plasmids with isolates of ST648 and ST131 to obtain an initial impression as to which virulence traits might be affected by ESBL-plasmid carriage. The differences in genetic content of the plasmids (Additional file 1: Figure S3) may harbor clues to explain the differences in the beneficial effects of plasmid carriage.

The onset of an infection by a pathogen begins with competition against endogenous microorganisms. Commensal strains are believed to act as protective microbiota which compete for both metabolic as well as niche space in the host [30]. We observed that all the strains were equally competitive, and had similar growth kinetics against both the commensal colicin-producing strains, except for the isolate PCV17433, which was out-competed in competition against IMT13858 (Fig. 1 and Additional file 1: Figure S2A–F). This observation suggests a protective role for the ESBL plasmid harbored by IMT17433 against colicin or competition, as both the wild type and re-transformed (T) variant showed similar growth. As both the competitors and wild type strains had similar metabolic capabilities, and all the variants showed similar capabilities to utilize different metabolic substrates, a role for metabolic competition seems unlikely. A similar observation was seen for competitive adhesion against both competitors in a porcine intestinal cell line. However, the PCV variant of IMT17887 belonging to ST648 showed lower colony counts in adhesion competition assays (Fig. 2), suggesting possible roles of ESBL-plasmids in competition or colicin protection.

Serum resistance is an important virulence characteristic of ExPEC. Serum resistance is mediated by large number of factors including the outer membrane proteins and plasmid associated factors [31, 32]. Phan et al. [26] demonstrated 56 genes which were essential for serum survival in EC958 (ST131) strain defining the serum resistome in ST131 dominant lineage. We observed that both PCV variants of IMT17433 and IMT17887 were sensitive to human serum, while PCV17433 was additionally sensitive to chicken serum (Fig. 3). As the ESBL-plasmid and sequence type vary between the two strains, and constituents of human and chicken serum are different this suggest that the PCV17887 variant might harbor additional serum resistance determinants. Nevertheless, these results suggest the possible involvement of ESBL plasmids in serum resistance. Earlier reports had shown that the introduction of ESBL-plasmid in the J53 conjugative strain increased its serum resistance, and ESBL-producing strains had higher serum resistance than non ESBL-producing strains [33, 34]. However, to our knowledge, we have shown for the first time enhanced serum resistance by transfer of an ESBL-plasmid into an isogenic background of the pandemic sequence types ST131 and ST648.

We observed the upregulation of factors like *ompA*, known to enhance serum resistance in K12 *E. coli* [27] and *nha*, *dnaJ*, *acrA*, which are the constituents of the serum resistome. Outer membrane proteins are one of the essential components, and are involved in many of the stress related defenses in pathogenic organisms. We observed that the PCV17443 and PCV17887 variants showed reduced viability and/growth in osmotic, salt, and acid stress. In none of the isolate variants did we observe an additional fitness cost due to plasmid carriage in isolates harboring an ESBL-plasmid. As the experiments were performed with unique triplets of wild type, plasmid-cured (PCV) and complemented (T) strains, the observed differences in phenotypes appear to be solely dictated by in presence of the respective plasmids.

## Conclusions

The inheritance of ESBL-plasmids in strains of the sequence types ST648 and ST131 does not lead to a general fitness burden in bacterial competition or serum resistance assays. For certain combinations of ESBL-plasmids with different bacterial chromosomal backgrounds, we observed that plasmids could confer beneficial virulence properties in some of strains of pandemic sequence types of ST131 and ST648.

## Additional file

**Additional file 1.** Additional Figures and Tables.

## References

[1] Pitout JD (2012). Extraintestinal pathogenic *Escherichia coli*: a combination of virulence with antibiotic resistance. Front Microbiol.

[2] Kaper JB, Nataro JP, Mobley HL (2004). Pathogenic *Escherichia coli*. Nat Rev Microbiol.

[3] Ranjan A (2017). Comparative genomics of *Escherichia coli* isolated from skin and soft tissue and other extraintestinal infections. MBio.

[4] Dho-Moulin M, Fairbrother JM (1999). Avian pathogenic *Escherichia coli* (APEC). Vet Res.

[5] Russo TA, Johnson JR (2003). Medical and economic impact of extraintestinal infections due to *Escherichia coli*: focus on an increasingly important endemic problem. Microb Infect.

[6] Shaikh S (2015). Antibiotic resistance and extended spectrum beta-lactamases: types, epidemiology and treatment. Saudi J Biol Sci.

[7] Mathers AJ, Peirano G, Pitout JD (2015). The role of epidemic resistance plasmids and international high-risk clones in the spread of multidrug-resistant Enterobacteriaceae. Clin Microbiol Rev.

[8] de Been M (2014). Dissemination of cephalosporin resistance genes between *Escherichia coli* strains from farm animals and humans by specific plasmid lineages. PLoS Genet.

[9] Ewers C (2014). CTX-M-15-D-ST648 *Escherichia coli* from companion animals and horses: another pandemic clone combining multiresistance and extraintestinal virulence?. J Antimicrob Chemother.

[10] Ranjan A (2015). Genomic and functional portrait of a highly virulent, CTX-M-15-producing H30-Rx subclone of *Escherichia coli* sequence type 131. Antimicrob Agents Chemother.

[11] Guenther S, Ewers C, Wieler LH (2011). Extended-spectrum beta-lactamases producing *E. coli* in wildlife, yet another form of environmental pollution?. Front Microbiol.

[12] Petty NK (2014). Global dissemination of a multidrug resistant *Escherichia coli* clone. Proc Natl Acad Sci USA.

[13] Clermont O (2009). Rapid detection of the O25b-ST131 clone of *Escherichia coli* encompassing the CTX-M-15-producing strains. J Antimicrob Chemother.

[14] Johnson JR, Johnston BD, Gordon DM (2017). Rapid and specific detection of the *Escherichia coli* sequence type 648 complex within phylogroup F. J Clin Microbiol.

[15] Nicolas-Chanoine MH, Bertrand X, Madec JY (2014). *Escherichia coli* ST131, an intriguing clonal group. Clin Microbiol Rev.

[16] Hirai I (2013). Detection of chromosomal blaCTX-M-15 in *Escherichia coli* O25b-B2-ST131 isolates from the Kinki region of Japan. Int J Antimicrob Agents.

[17] Rodriguez I (2014). Chromosomal location of blaCTX-M genes in clinical isolates of *Escherichia coli* from Germany, The Netherlands and the UK. Int J Antimicrob Agents.

[18] Andersson DI, Hughes D (2010). Antibiotic resistance and its cost: is it possible to reverse resistance?. Nat Rev Microbiol.

[19] Crossman LC (2010). A commensal gone bad: complete genome sequence of the prototypical enterotoxigenic *Escherichia coli* strain H10407. J Bacteriol.

[20] Schaufler K (2016). Carriage of extended-spectrum beta-lactamase-plasmids does not reduce fitness but enhances virulence in some strains of pandemic *E. coli* lineages. Front Microbiol.

[21] Schaufler K (2013). ESBL-plasmids carrying toxin-antitoxin systems can be “cured” of wild-type *Escherichia coli* using a heat technique. Gut Pathog.

[22] Bano S (2013). Pattern of induction of colicin E9 synthesis by sub MIC of Norfloxacin antibiotic. Microbiol Res.

[23] Shubin M (2016). Identifying multiple potential metabolic cycles in time-series from biolog experiments. PLoS ONE.

[24] Nandanwar N (2014). Extraintestinal pathogenic *Escherichia coli* (ExPEC) of human and avian origin belonging to sequence type complex 95 (STC95) portray indistinguishable virulence features. Int J Med Microbiol.

[25] Wang Y (2002). The function of OmpA in *Escherichia coli*. Biochem Biophys Res Commun.

[26] Phan MD (2013). The serum resistome of a globally disseminated multidrug resistant uropathogenic *Escherichia coli* clone. PLoS Genet.

[27] Weiser JN, Gotschlich EC (1991). Outer membrane protein A (OmpA) contributes to serum resistance and pathogenicity of *Escherichia coli* K-1. Infect Immun.

[28] Rupp ME, Fey PD (2003). Extended spectrum beta-lactamase (ESBL)-producing Enterobacteriaceae: considerations for diagnosis, prevention and drug treatment. Drugs.

[29] Ranjan A (2016). Molecular epidemiology and genome dynamics of New Delhi metallo-beta-lactamase (NDM) producing extraintestinal pathogenic *E. coli* (ExPEC) strains from India. Antimicrob Agents Chemother.

[30] Ubeda C, Djukovic A, Isaac S (2017). Roles of the intestinal microbiota in pathogen protection. Clin Transl Immunol.

[31] Sukupolvi S (1992). Plasmid-mediated serum resistance in *Salmonella enterica*. Microb Pathog.

[32] Leying H (1990). The capsular polysaccharide is a major determinant of serum resistance in K-1-positive blood culture isolates of *Escherichia coli*. Infect Immun.

[33] Hussain A (2012). Multiresistant uropathogenic *Escherichia coli* from a region in India where urinary tract infections are endemic: genotypic and phenotypic characteristics of sequence type 131 isolates of the CTX-M-15 extended-spectrum-beta-lactamase-producing lineage. Antimicrob Agents Chemother.

[34] Sahly H (2004). Increased serum resistance in *Klebsiella pneumoniae* strains producing extended-spectrum beta-lactamases. Antimicrob Agents Chemother.

