# Supplementary material for: ESBL-plasmid carriage in E. coli enhances in vitro bacterial competition fitness and serum resistance in some strains of pandemic sequence types without overall fitness cost
Source: Gut Pathog. 2018 Jun 15;10:24. doi: 10.1186/s13099-018-0243-z (PMC6003029; doi:10.1186/s13099-018-0243-z)
Supplement: Supplementary file 1 — Additional file 1. Additional Figures and Tables. [file 13099_2018_243_MOESM1_ESM.pdf]

Figure S1

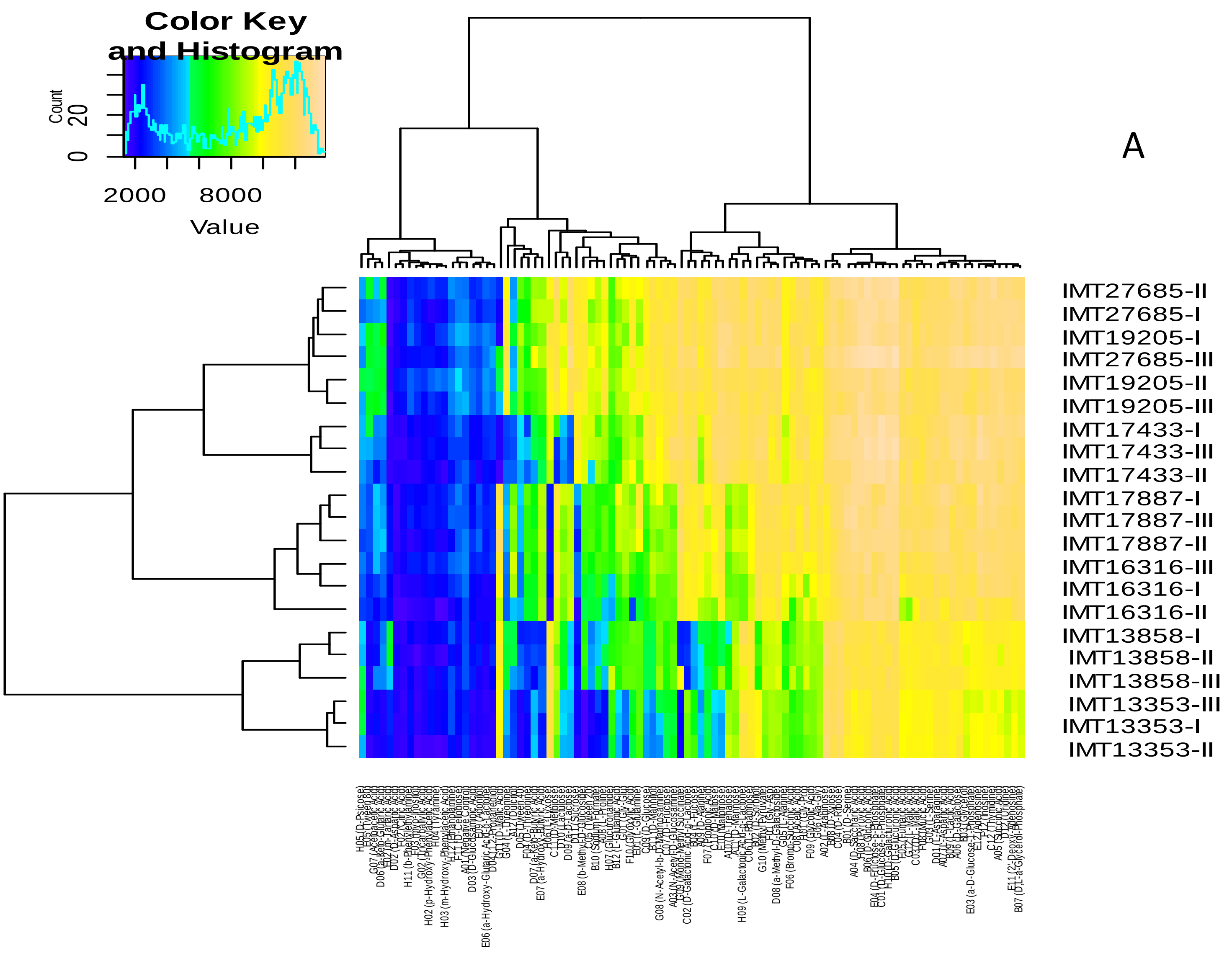

Heat maps generated using R-program for PM1 (carbon sources) for all seven strains including five ESBL strains and two competitors. Horizontal panel displays the names of metabolite and vertical panel depicts the strains. The color represent the extent of utilization as denoted by color key on top.

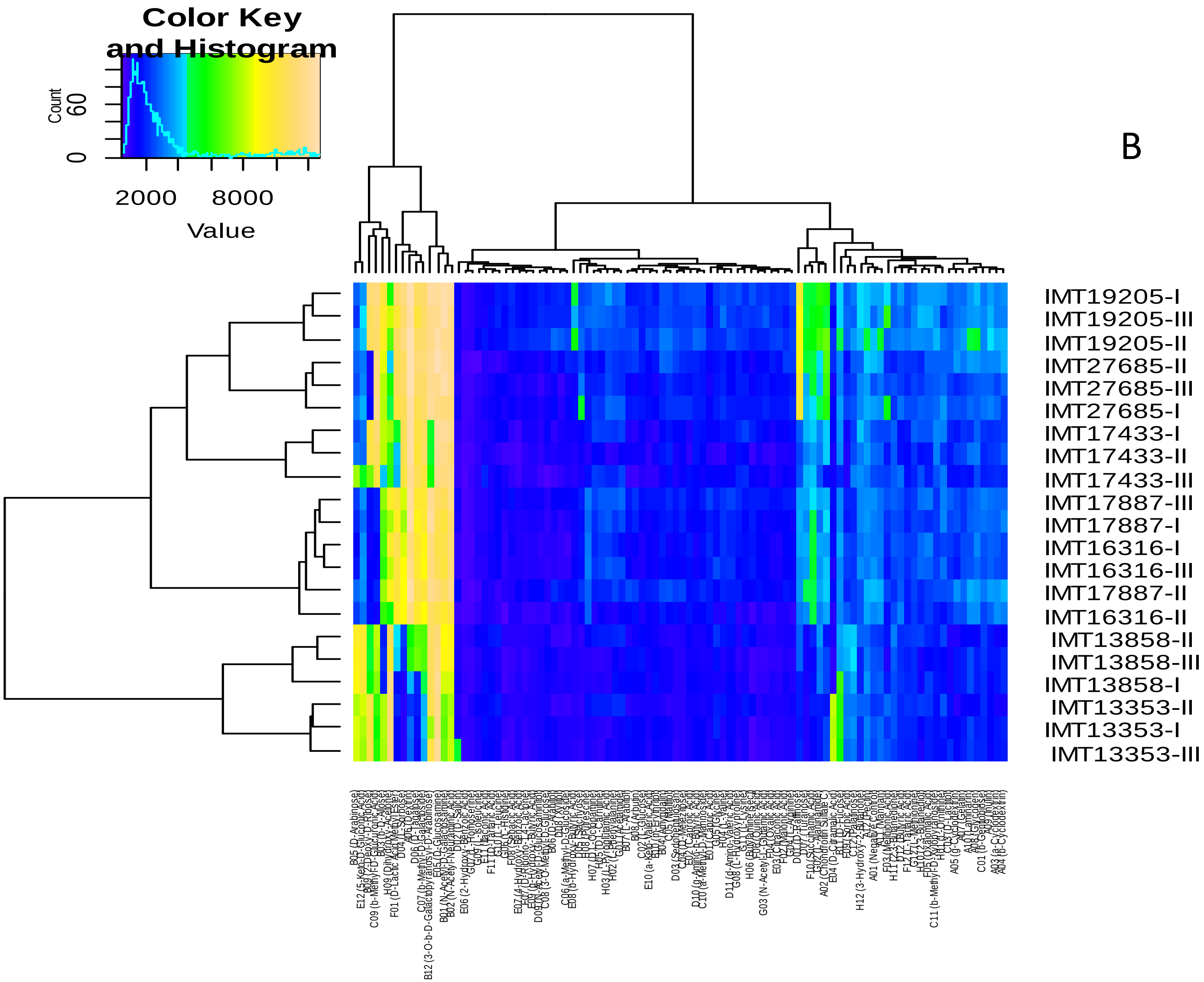

Heat maps generated using R-program for PM2 (carbon sources) for all seven strains including five ESBL strains and two competitors. Horizontal panel displays the names of metabolite and vertical panel depicts the strains. The color represent the extent of utilization as denoted by color key on top.

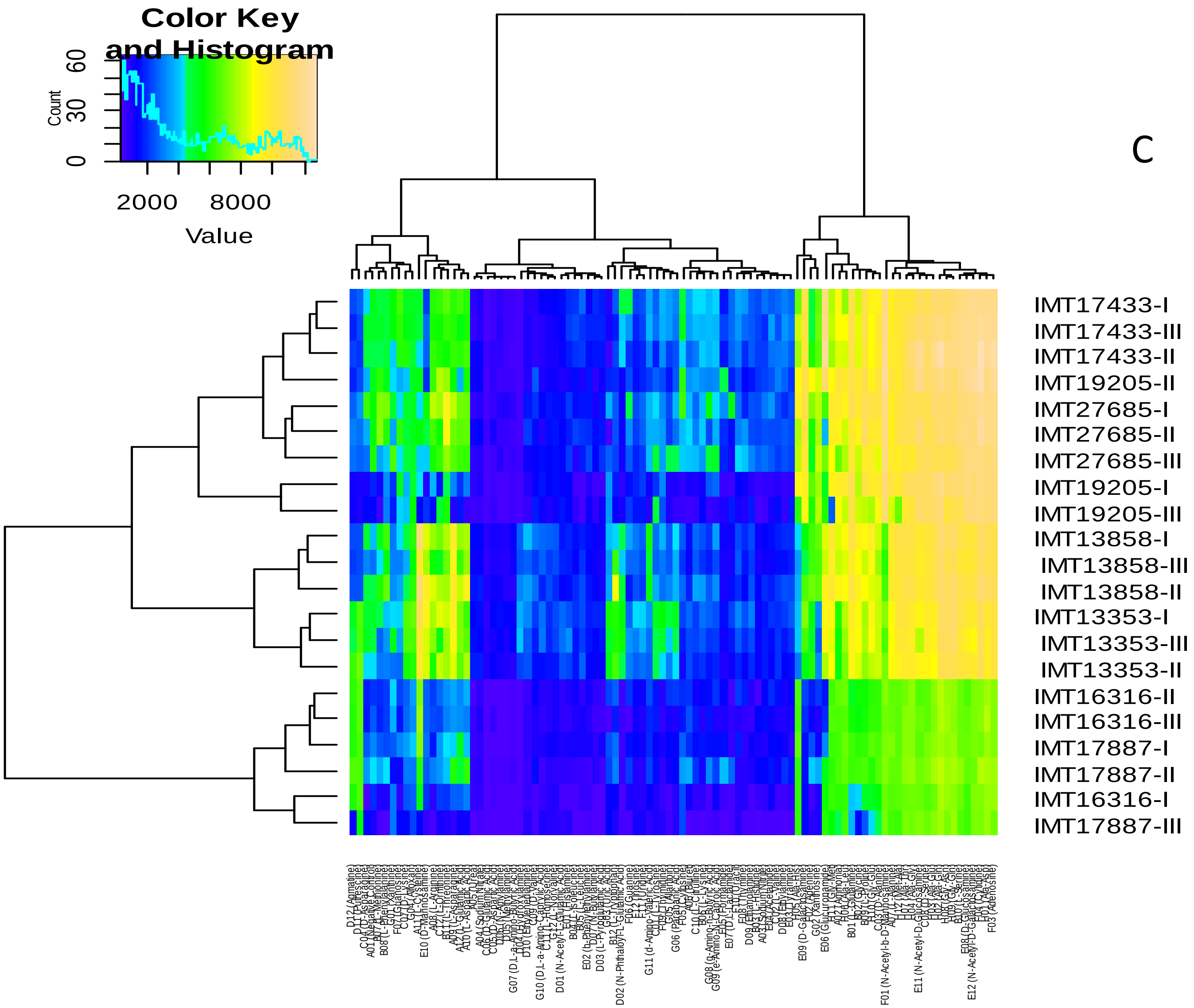

Heat maps generated using R-program for PM3 (Nitrogen sources) for all seven strains including five ESBL strains and two competitors. Horizontal panel displays the names of metabolite and vertical panel depicts the strains. The color represent the extent of utilization as denoted by color key on top.

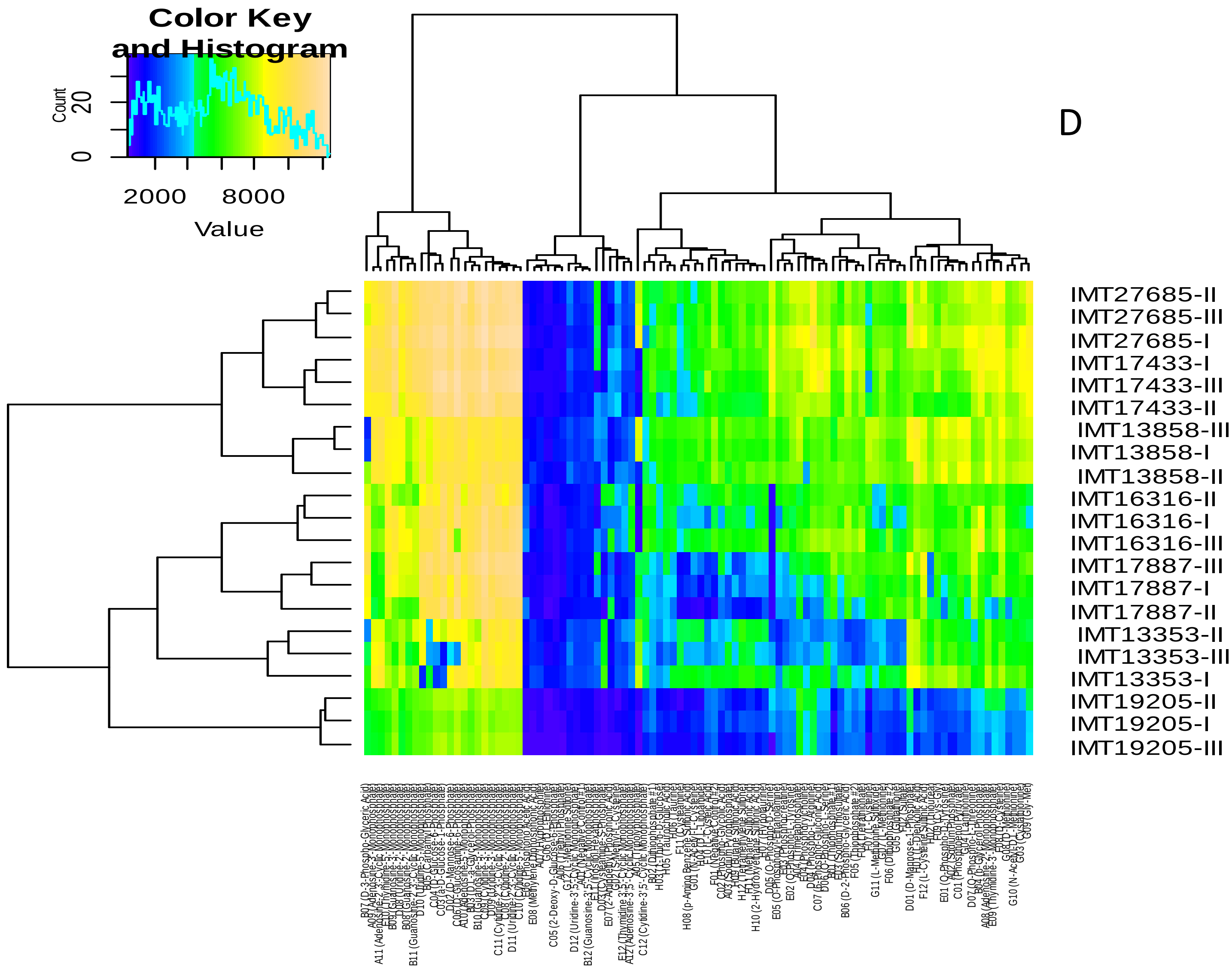

Heat maps generated using R-program for PM4 (phosphorous and sulphur sources) for all seven strains including five ESBL strains and two competitors. Horizontal panel displays the names of metabolite and vertical panel depicts the strains. The color represent the extent of utilization as denoted by color key on top.

Figure S2

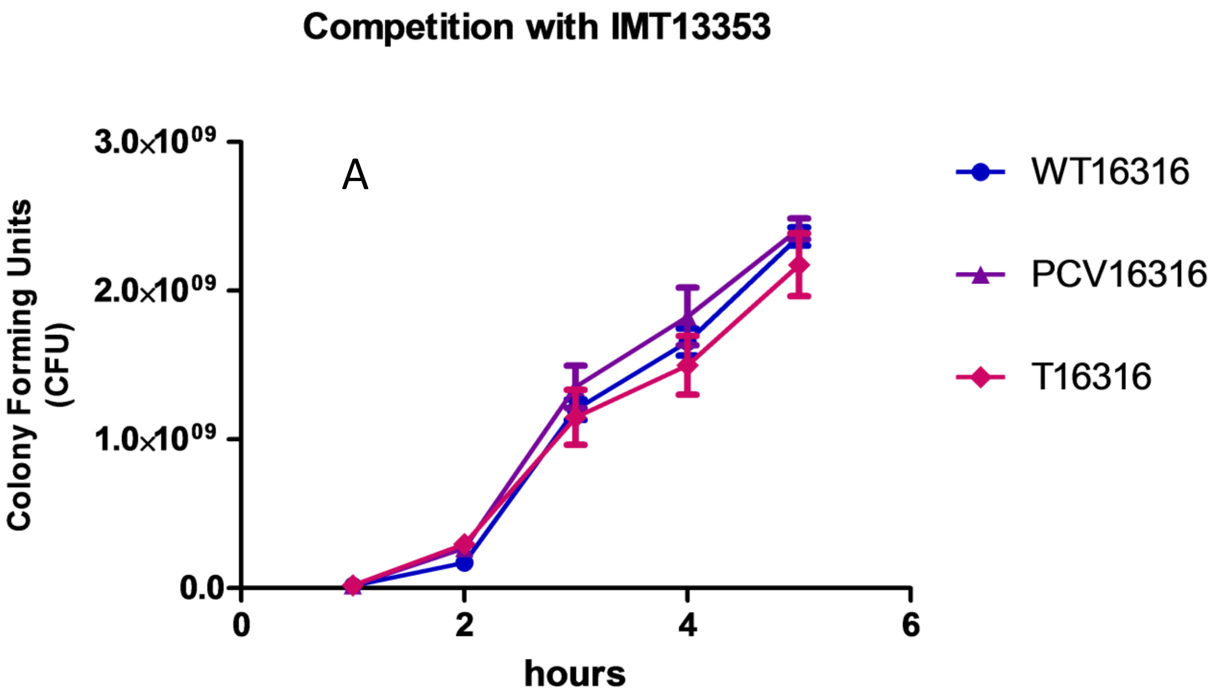

Competition assay between variants of IMT16316 and IMT13353 for five hours. Growth is measured in terms of colony forming units

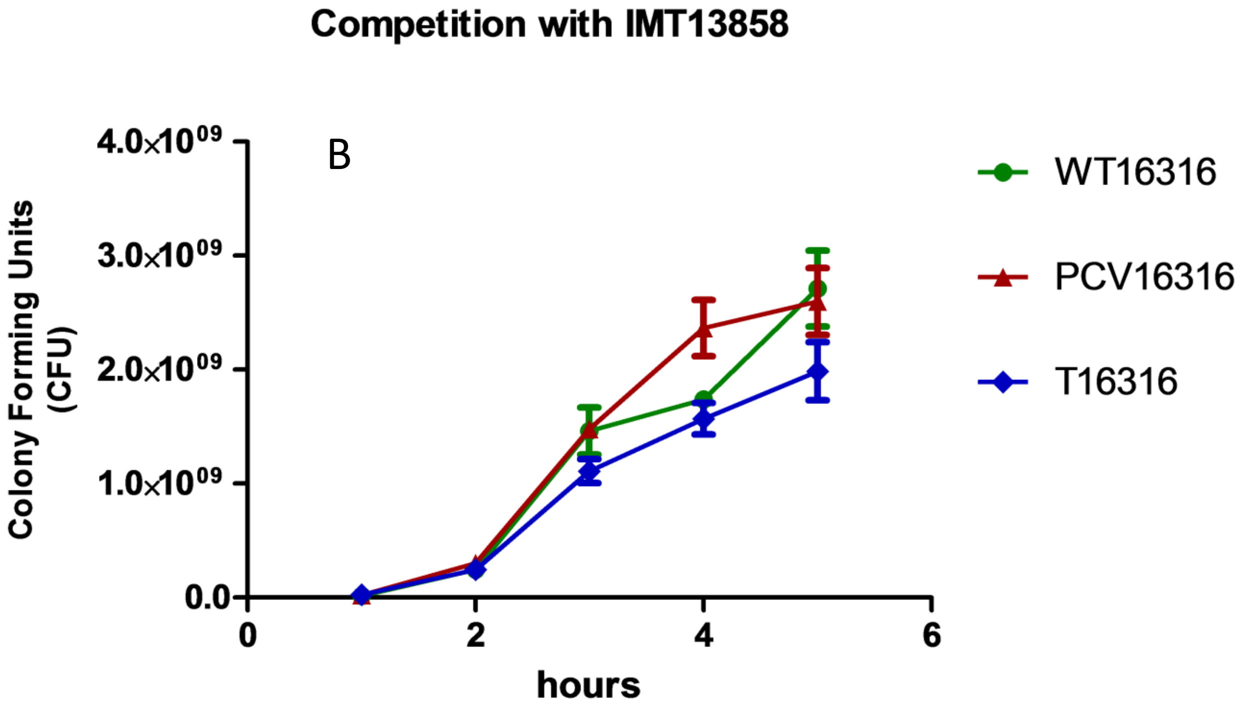

Competition assay between variants of IMT16316 and IMT13858 for five hours. Growth is measured in terms of colony forming units

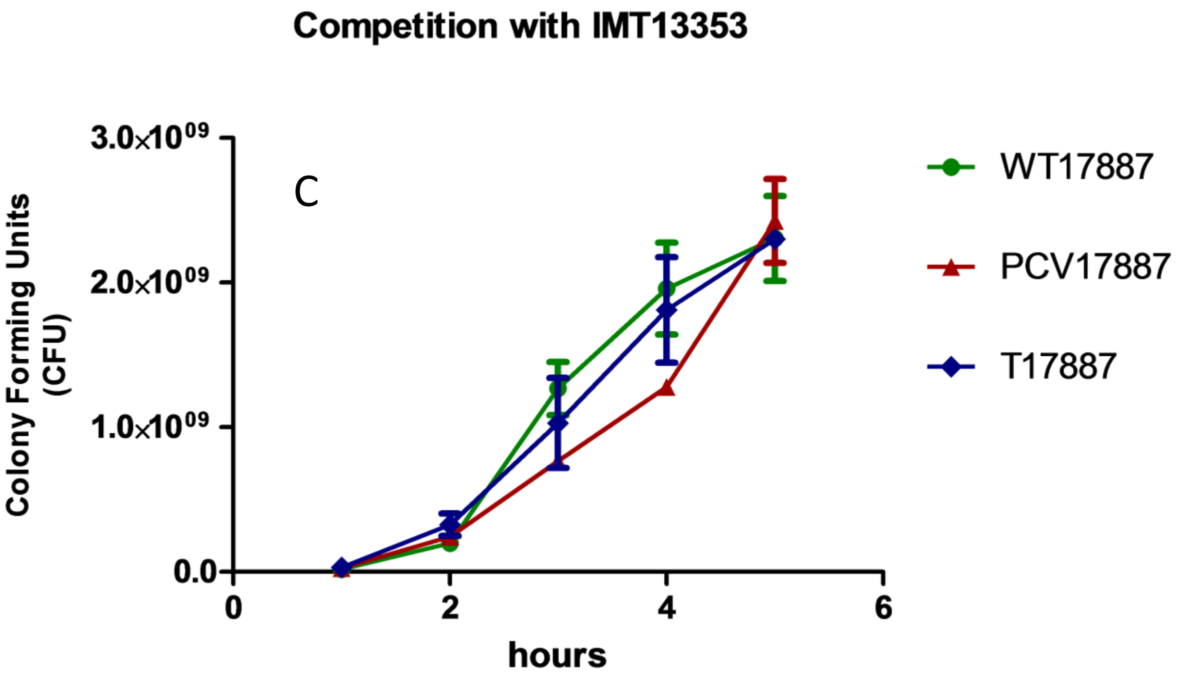

Competition assay between variants of IMT17887 and IMT13353 for five hours. Growth is measured in terms of colony forming units

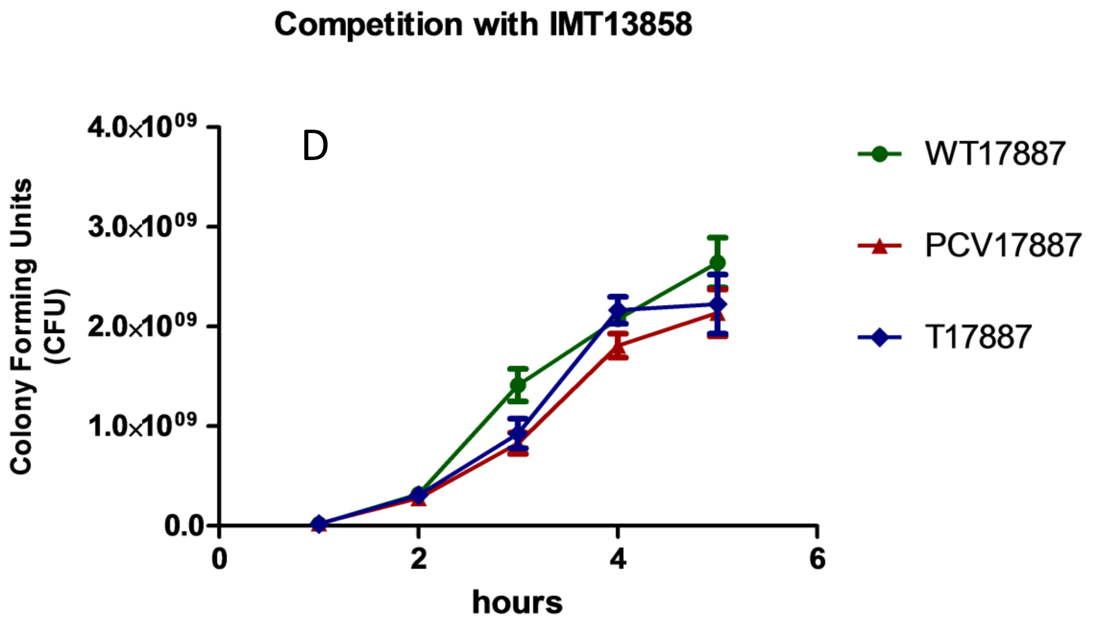

Competition assay between variants of IMT17887 and IMT13858 for five hours. Growth is measured in terms of colony forming units

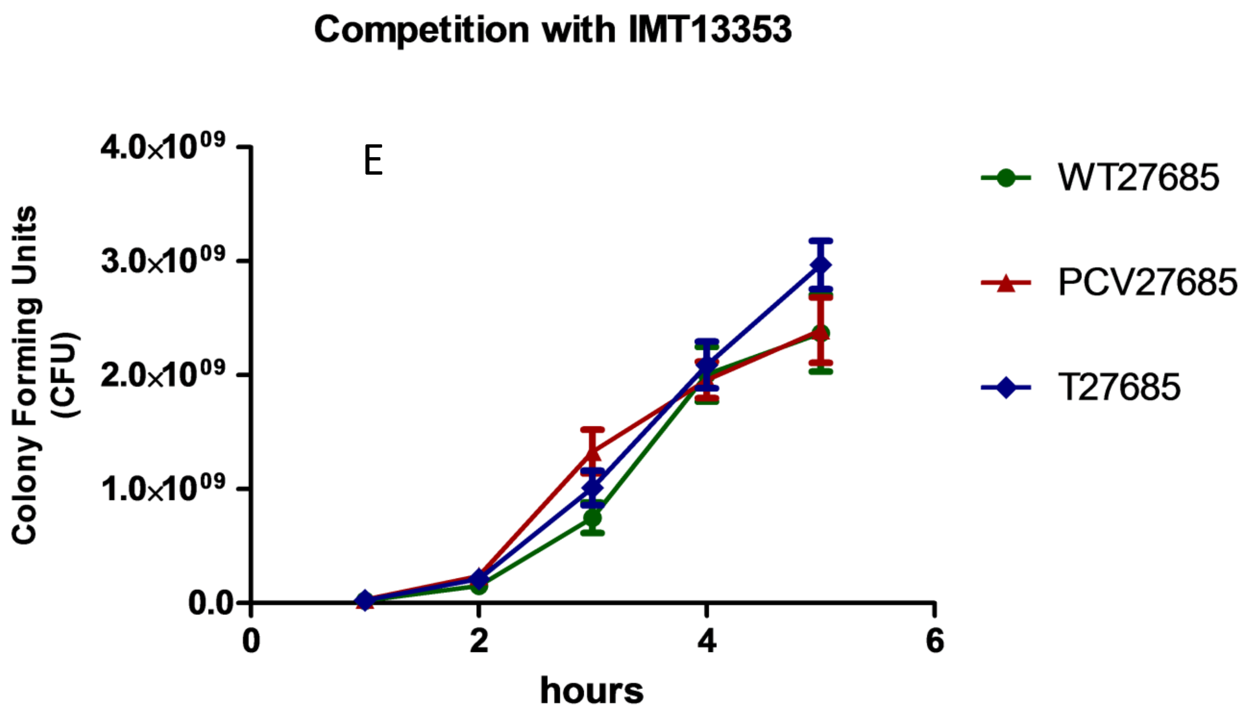

Competition assay between variants of IMT27685 and IMT13353 for five hours. Growth is measured in terms of colony forming units

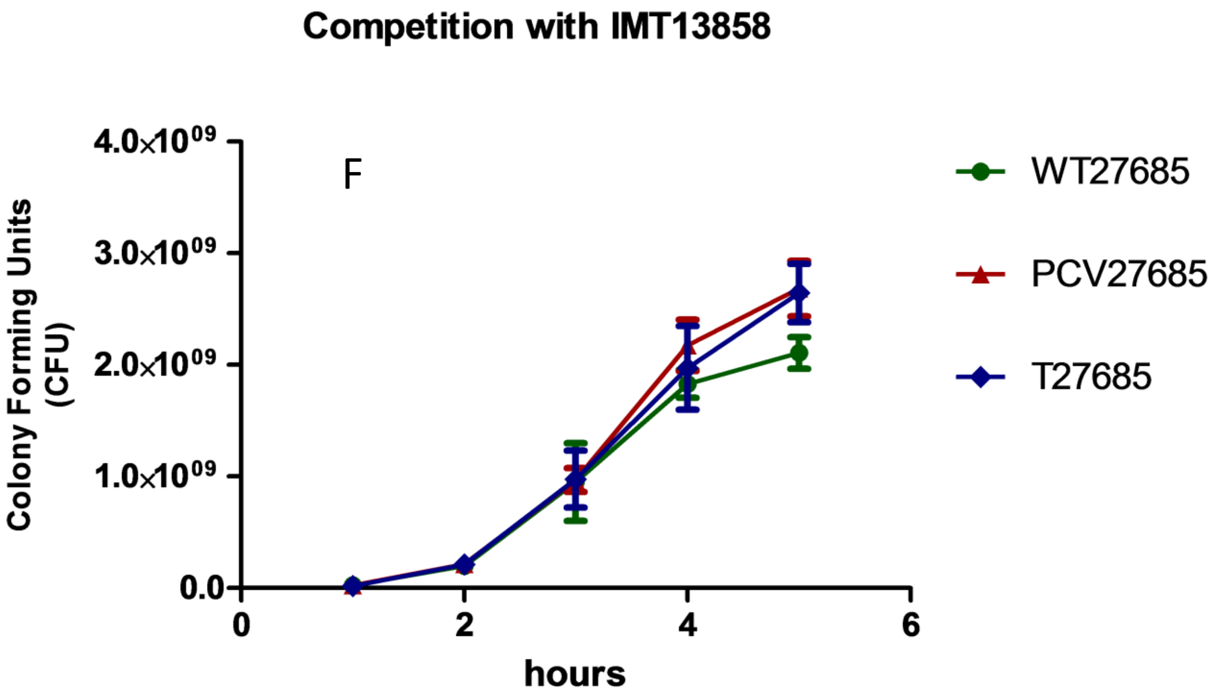

Competition assay between variants of IMT27685 and IMT13858 for five hours. Growth is measured in terms of colony forming units

Figure S3

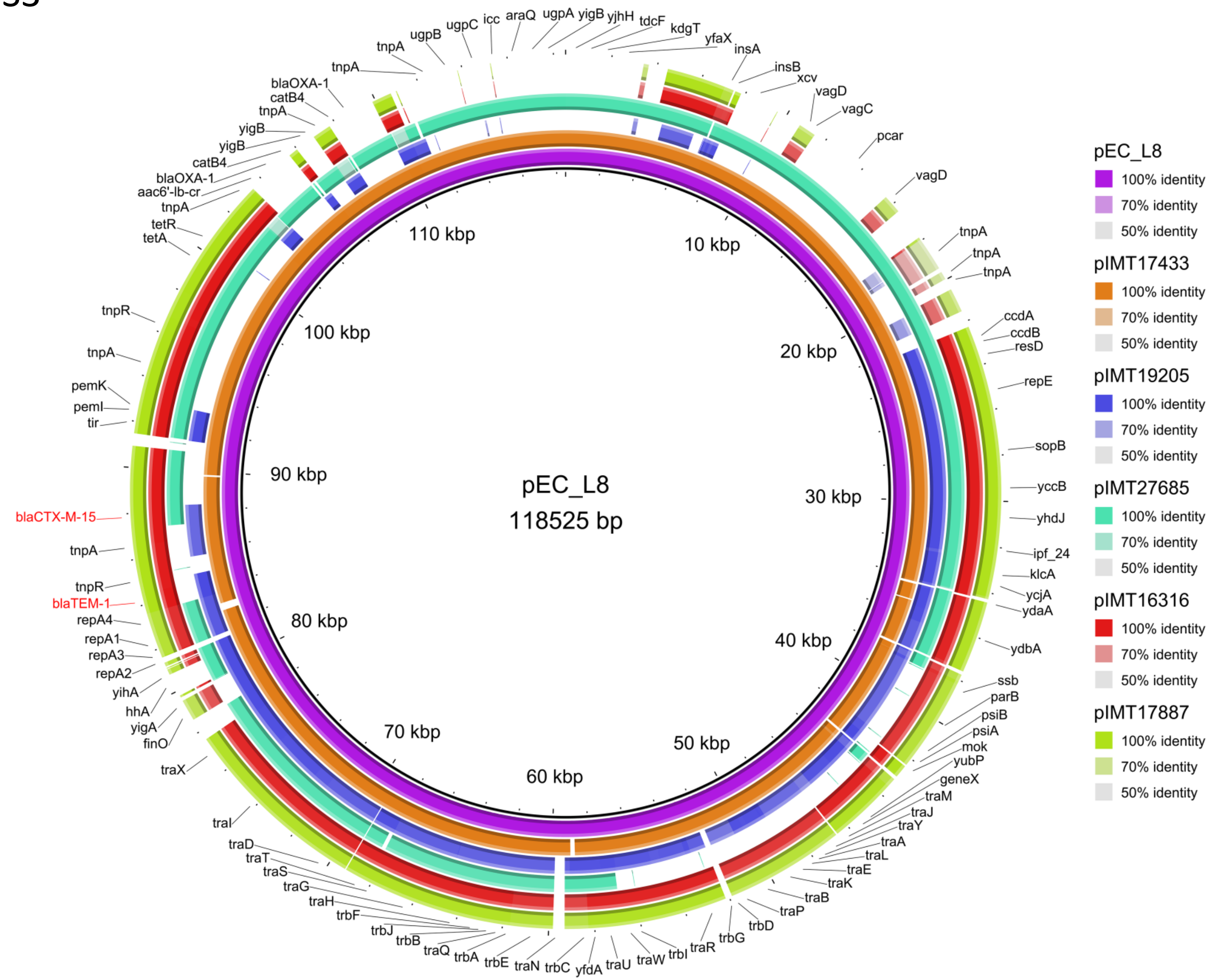

BRIG image of the ESBL-plasmids of the strains in the study, pEC_L8 served as reference for the comparison while other representative concentric rings represent the ESBL-plasmids as pIMT17433, pIMT19205, pIMT27685, pIMT 16316 and pIMT17887 respectively.

Table S1: Details of the strains used in this study and their characteristics

Strain	Host	Origin	ESBL type	ESBL-plasmid REL/RIP proteins and Inc group	ESBL-plasmid size (bp)	Number of additional plasmids (sizes in bp)	Sequence types
IMT17433	Dog (*C. lupus familiaris*)	Urinary tract infection	CTX-M-15	MOB_{F12}, RepFIA/FII, IncF	122,823	1 (1600)	131
IMT19205	Brown rat (*R. norvegicus*)	Feces	CTX-M-27	MOB_{F12}, RepFIA/FIB/FII , IncF	166,151	2 (1500, 5200)	131
IMT27685	Raven (*C. corax*)	Feces	CTX-M-15	MOB_{F12}, RepFIA/FII, IncF	133,611	1 (1600)	131
IMT16316	Blackbird (*T. merula*)	Feces	CTX-M-15	MOB_{F12}, RepFIA/FIB, IncF	136,508	4 (2500, 3200, 4100, 7000)	648
IMT17887	Horse (*E. ferus caballus*)	Soft tissue/wound infection	CTX-M-15	MOB_{F12}, RepFIA/FIB, IncF	143,494	2 (2600, 7100)	648
